# Segmenting computed tomograms for cardiac ablation using machine learning leveraged by domain knowledge encoding

**DOI:** 10.3389/fcvm.2023.1189293

**Published:** 2023-10-02

**Authors:** Ruibin Feng, Brototo Deb, Prasanth Ganesan, Fleur V. Y. Tjong, Albert J. Rogers, Samuel Ruipérez-Campillo, Sulaiman Somani, Paul Clopton, Tina Baykaner, Miguel Rodrigo, James Zou, Francois Haddad, Matei Zahari, Sanjiv M. Narayan

**Affiliations:** ^1^ Department of Medicine and Cardiovascular Institute, Stanford University, Stanford, CA, United States; ^2^ Heart Center, Department of Clinical and Experimental Cardiology, Amsterdam UMC, University of Amsterdam, Amsterdam, Netherlands; ^3^ Bioengineering Department, University of California, Berkeley, Berkeley, CA, United States; ^4^ CoMMLab, Universitat Politècnica de València, Valencia, Spain; ^5^ Department of Biomedical Data Science, Stanford University, Stanford, CA, United States; ^6^ Department of Computer Science, Stanford University, Stanford, CA, United States

**Keywords:** cardiac CT segmentation, machine learning, mathematical modeling, domain knowledge, atrial fibrillation, ablation

## Abstract

**Background:**

Segmentation of computed tomography (CT) is important for many clinical procedures including personalized cardiac ablation for the management of cardiac arrhythmias. While segmentation can be automated by machine learning (ML), it is limited by the need for large, labeled training data that may be difficult to obtain. We set out to combine ML of cardiac CT with domain knowledge, which reduces the need for large training datasets by encoding cardiac geometry, which we then tested in independent datasets and in a prospective study of atrial fibrillation (AF) ablation.

**Methods:**

We mathematically represented atrial anatomy with simple geometric shapes and derived a model to parse cardiac structures in a small set of *N* = 6 digital hearts. The model, termed “virtual dissection,” was used to train ML to segment cardiac CT in *N* = 20 patients, then tested in independent datasets and in a prospective study.

**Results:**

In independent test cohorts (*N* = 160) from 2 Institutions with different CT scanners, atrial structures were accurately segmented with Dice scores of 96.7% in internal (IQR: 95.3%–97.7%) and 93.5% in external (IQR: 91.9%–94.7%) test data, with good agreement with experts (*r* = 0.99; *p* < 0.0001). In a prospective study of 42 patients at ablation, this approach reduced segmentation time by 85% (2.3 ± 0.8 vs. 15.0 ± 6.9 min, *p* < 0.0001), yet provided similar Dice scores to experts (93.9% (IQR: 93.0%–94.6%) vs. 94.4% (IQR: 92.8%–95.7%), *p* = NS).

**Conclusions:**

Encoding cardiac geometry using mathematical models greatly accelerated training of ML to segment CT, reducing the need for large training sets while retaining accuracy in independent test data. Combining ML with domain knowledge may have broad applications.

## 1. Introduction

Segmentation of cardiac imaging data is central to several aspects of clinical care, but can be challenging and time consuming. This may hinder the development of large reference databases. In atrial fibrillation (AF), early rhythm control by ablation reduces morbidity and mortality (1), yet segmenting computed tomography (CT) for ablation by annotating the left atrium, pulmonary veins (PVI) and other target sites (2) still requires substantial human intervention even with current cardiac mapping systems (3), which can be time consuming and introduce errors (4).

Machine learning (ML) can automate image segmentation (5). However, one of the biggest challenge in ML applications is the lack of large annotated ground truth data sets identified by LeCun and others (5). This issue is particularly critical in medicine and healthcare applications (6–8) due to technical, privacy, and regulatory concerns. Many publicly available labeled datasets contain ∼100 cases (9–11), yet traditional ML studies typically use large cohorts (∼70 cases) for training and thus test in only ∼30 cases (12–15), which may limit generalizability and hinder wider application (16, 17).

Methods such as transfer learning showed advances in alleviating the need for large training datasets (18, 19). However, many are tailored for medical image classification instead of segmentation (20) or exhibit inconsistent segmentation performance across tasks and datasets (21). Other techniques such as synthetic data generation (22) and data augmentation (23) can artificially enlarge training sets, but risk lacking real-world diversity (24) or introducing bias due to overfitting (25). Indeed, atlases that leverage anatomic knowledge have long been used for image segmentation (26, 27), but their performance will be compromised when faced with anatomic variants unrepresented in the training data (28).

**Figure 1:**
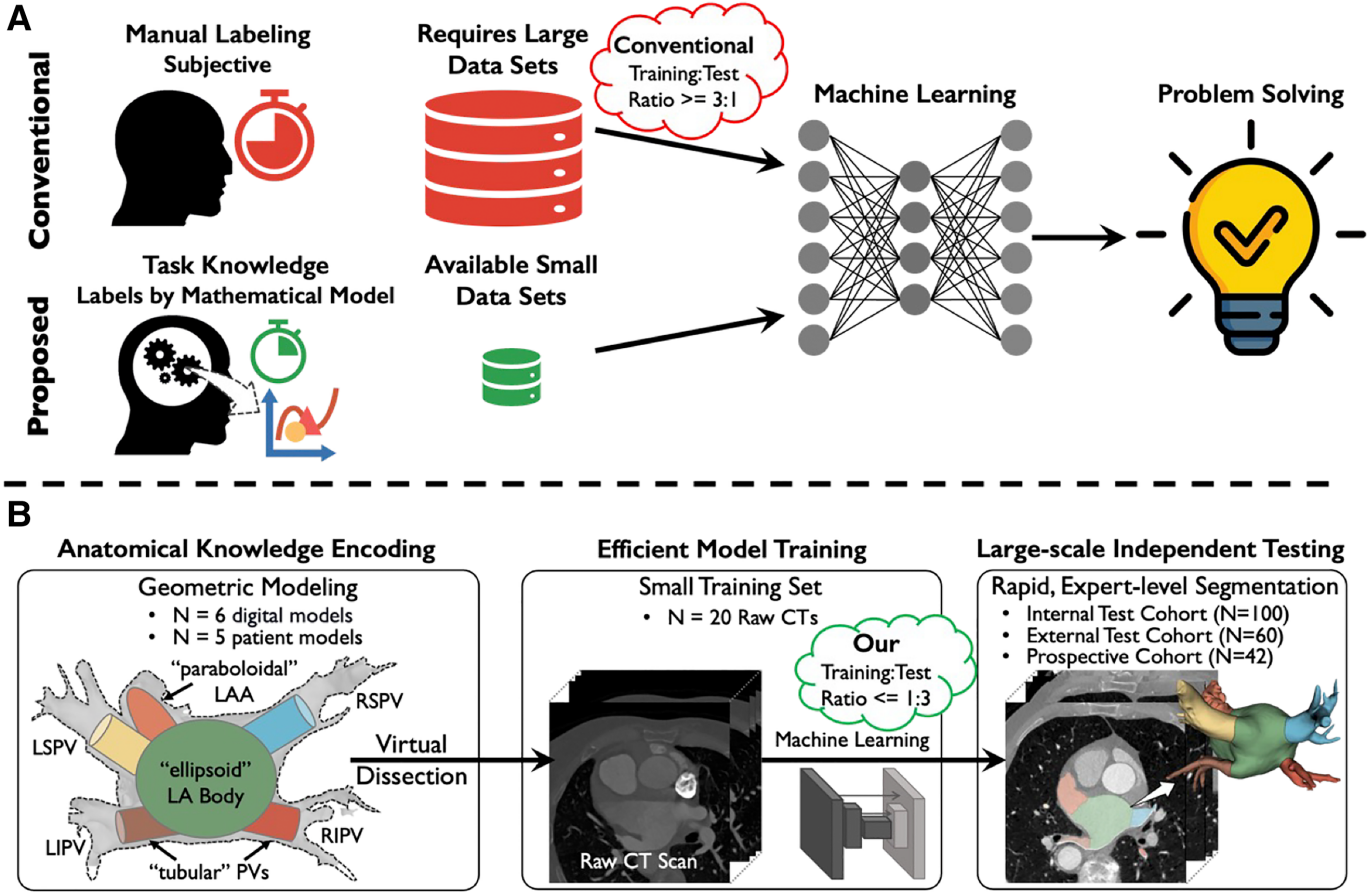
Concept and overview. (**A**) Conventional machine learning (top) can learn patterns in complex data, but requires laborious manual labeling, in large datasets which may be difficult to obtain. Conversely, our proposed approach (bottom) used natural intelligence to replace manual labeling with anatomical concepts encoded mathematically of domain knowledge, to learn rapidly from small datasets. (**B**) We applied mathematical encoding to segment heart CT scans via ML of small datasets. We represented heart structures as geometric primitives (“virtual dissection”). This was used to train ML on a small dataset (*N* = 20) and was able to accurately segment hearts in 2 larger cohorts from different institutions (*N* = 100, 60). In a prospective study (*N* = 42), the model segmented cardiac CT scans faster, but as accurately as experts. Acronyms: LA, left atrium; LSPV, left superior pulmonary vein; LIPV, left inferior pulmonary vein; RSPV, right superior pulmonary vein; RIPV, right inferior pulmonary vein; LAA, left atrial appendage.

One novel approach is to train ML with conceptual domain (expert) knowledge to potentially reduce the need for massive amounts of data for training (29, 30) (Figure 1A), analogous to how humans learn (30). Lake et al. used this approach to generate handwritten characters with human-level performance from 1 exemplar, by parsing characters into simple primitives that were composited to create new characters (31). However, domain knowledge for medical applications is rarely sufficient to reduce training sizes for ML (32, 33).

We hypothesized that ML models could be trained using very small datasets if combined with some mathematical knowledge of the task at hand, or *domain knowledge encoding*. Specifically, we developed mathematical digital models of the cardiac anatomy (the atria) from generic publicly available databases. While we had access to a large dataset of 232 cases, we leveraged domain knowledge to train ML models in a deliberately small cohort, setting aside more cases for testing from 2 large independent datasets. We also tested our model prospectively in a clinical study (Figure 1B).

## 2. Materials and methods

Figure 1B outlines our study design, containing the following steps: (1) We developed algorithms that encode atrial and pulmonary vein anatomies; (2) The algorithm was used to train ML to segment cardiac CT, using a small development cohort; (3) The trained ML was tested in 2 external cohorts from different institutions; (4) The combined domain encoded/ML model was tested prospectively to segment CTs in patients undergoing AF ablation, compared to a panel of 3 experts.

### 2.1. Development and testing clinical cohort

We identified *N* = 130 consecutive patients who had undergone AF ablation, had CT scans at Stanford Health Care from October 2014 to July 2019, each of whom provided written informed consent. We split this data set randomly into *N* = 30 for algorithm deriving and model training (*Development* cohort), and *N* = 100 patients as a hold-out cohort (*Internal Test* cohort). To further evaluate our approach, we utilized an external publicly available dataset [MM-WHS (10), *N* = 60] from a different institution and different CT scanners (*External Test* cohort).

### 2.2. Deriving virtual dissection algorithm

**Figure 2:**
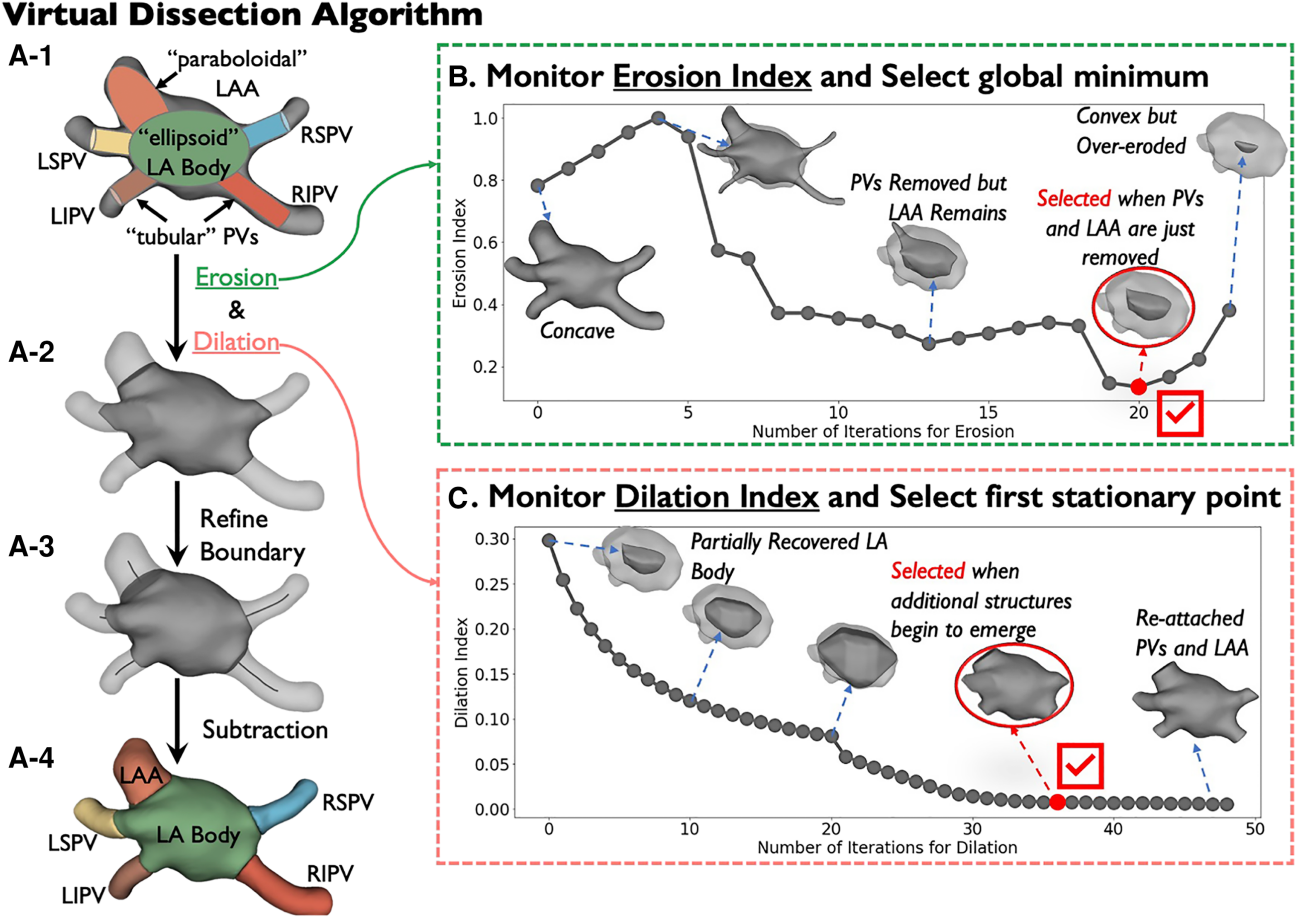
Virtual Dissection algorithm. (**A**) The detailed pipeline. (**B**) The progress of the iterative erosion. The automatically selected iteration for erosion is highlighted in red. (**C**) The progress of the iterative dilation. The automatically selected iteration for dilation is highlighted in red. Acronyms: LA, left atrium; LSPV, left superior pulmonary vein; LIPV, left inferior pulmonary vein; RSPV, right superior pulmonary vein; RIPV, right inferior pulmonary vein; LAA, left atrial appendage.

We derived our mathematical encoding model from *N* = 6 publicly available 3D heart models (Figure 2A-1), built using Gaussian process morphable models (34). We employed these digital models solely as simplified yet accurate templates to facilitate the development, analysis, and tuning of our algorithm.

Inspired by computer graphics (CG) modeling, this “virtual dissection” method identifies critical structures using mathematical encoding (Figure 2). CG uses simple geometrical shapes (‘primitives’) to represent complex objects such as human bodies, that form the basis for techniques such as kinematic modeling that learns 3D human poses from YouTube videos (35) to generate animations. We represented the left atrium (LA) as an ellipsoid; pulmonary veins (PVs) as circular cylinders; and left atrial appendage (LAA) as a paraboloid (Figure 2A-1).

We then reasoned that heart structures can be geometrically parsed by separating the ellipsoidal convex LA from the complex concave whole heart. We used mathematical erosion, dilation (36) and subtraction for this purpose (Figure 2A). First, we dissected digital shells by a novel application of erosion of concave junctions between tubular PVs and paraboloidal LAA with the ellipsoidal left atrium. We propose an *Erosion Index*, which indicates erosion progression toward a convex shape and can be used to identify the optimal number of erosion iterations (Figure 2B). We then applied dilation to ensure the LA retained its original size after erosion and introduced a *Dilation Index* to track the restoration process, which helps determine when to stop dilation before PVs and LAA are re-attached (Figure 2C).

To encode the variability of LA geometries across patients, we optimized the virtual dissection algorithm using small clinical seed data from N={0,5,10,20,30} patients in the *Development* cohort. We trained support vector machines (SVMs) with manually segmented images in patients from the seed sets to predict the optimal number of erosion and dilation iterations.

**Figure 3:**
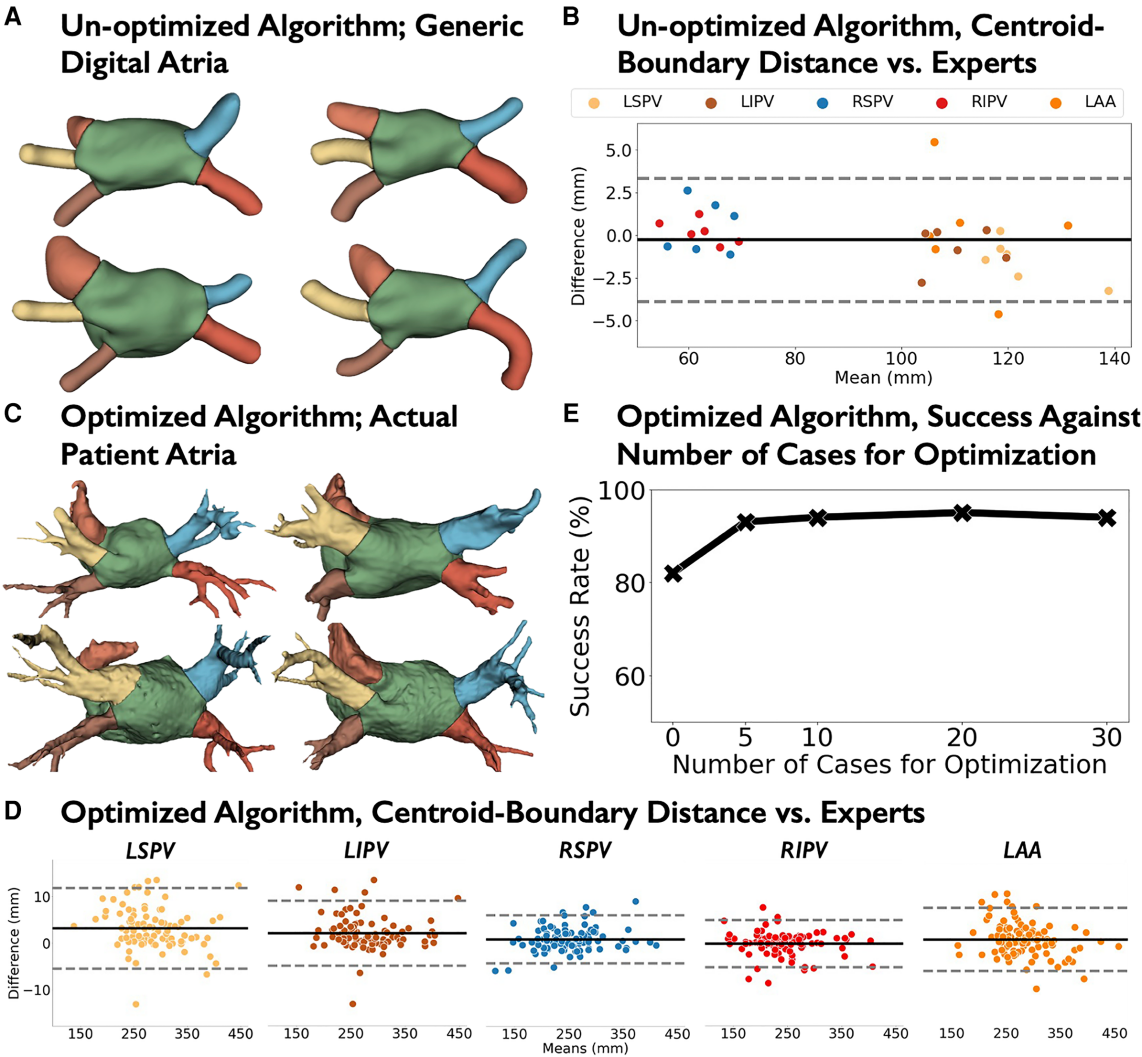
Virtual dissection performance. (**A**) Representative samples of digital atria geometrically parsed by un-optimized algorithm. (**B**) Bland-Altman plots of the centroid-to-boundary of un-optimized algorithm vs. experts in 6 digital atria. After optimizing Virtual Dissection with *N* = 5 patient cases from the development cohort, (**C**) Representative patient atria from optimized algorithm in independent Test cohort (*N* = 100). (**D**) Bland–Altman plots of the centroid-to-boundary distance of optimized algorithm vs. experts in the Test cohort. (**E**) Success rate of virtual dissection algorithm using *N*={0, 5, 10, 20, 30} cases. Acronyms: LSPV, left superior pulmonary vein; LIPV, left inferior pulmonary vein; RSPV, right superior pulmonary vein; RIPV, right inferior pulmonary vein; LAA, left atrial appendage.

After the left atrium body is isolated after erosion and dilation, we refined boundaries between the LA body and the PVs and LAA (Figure 2A-3) by calculating centerlines from the LA centroid to the centroid of each virtually dissected structure using the Voronoi diagram (37), a method previously used in aorta and great vessels segmentation (9, 38, 39). The original boundaries from the erosion-dilation phase were then refined using a plane aligned perpendicularly to these centerlines. Accuracy of virtual dissection was assessed by centroid-boundary distance and other metrics outlined below in Statistical Analysis.

### 2.3. Small cohorts of virtually dissected atria can train ML for CT segmentation

We used virtually dissected atria of *N* = 20 patients from the *Development* cohort to train ML to segment chest CT scans. We trained a deep neural network architecture, nnU-Net (Supplementary Figure 1), which has been widely used in 23 public datasets (40). For training, we normalized then augmented raw CT scans as input, with the virtual dissected atria as ground truths. We ensured similar voxel spacing for test and training samples. The training protocol is detailed in Supplementary Methods. We applied the trained ML to the independent *Internal Test* and the *External Test* cohorts, neither of which was used for training. Accuracy of ML segmentation was assessed by Dice similarity coefficient and other metrics outlined below in Statistical Analysis.

### 2.4. Prospective study

We prospectively compared our ML approach to experts for segmenting cardiac CT scans in patients prior to AF ablation. The primary endpoints were annotation time and accuracy as assessed by Dice similarity coefficient. The study was approved by the review board of Stanford University Human Subjects Protection Committee, and all subjects gave written informed consent (NCT02997254).

Patient entry criteria were patients undergoing ablation for symptomatic AF refractory to at least one anti-arrhythmic medication between January 1st, 2022, and March 30th, 2022 (*N* = 96). The predefined exclusion criteria were (1) no valid DICOM files (25 cases), (2) CT performed >90 days before ablation (21 cases), and (3) with LAA closure procedures (8 cases). We identified *N* = 42 consecutive patients (Prospective cohort). CT images in our prospective study were scanned using the third-generation dual-source CT system (Somatom Force; Siemens AG). The CT images had axial sections of 0.7 mm thickness and typical in-plane pixel size of 0.42 × 0.42 mm.

A panel of 3 experts manually annotated raw CT scans with 3D slicer (41) independently. Each expert had first practiced on several run-in cases, separate from the study cohort, to become familiar with the workflow. During annotation, a bounding box was initially created to identify the LA (including the main branches of PVs and LAA). Several foreground/background seeds were added to these regions, and the region-growing algorithm was applied to get the initial LA geometry. Manual corrections were performed as needed with no further constraints. The final LA segmentation was smoothed using default parameters and exported as a NIFTI file for evaluation. The time taken from loading the CT to exporting the file was recorded for comparison.

### 2.5. Statistical analysis

We utilized a newly designed metric, the centroid-boundary distance, along with two standard metrics for segmentation tasks (9–15)—Dice similarity coefficient and average surface distance, to evaluate our model's accuracy in capturing 2D LA-PV/LAA boundaries, the global 3D structures, and the local 3D shapes and contours, respectively. Mathematically, the centroid-boundary distance is calculated as the average of all the distances from the centroid of the heart to points on the LA-PV/LAA boundary. The Dice similarity score measures spatial overlap between the model prediction and the ground truth, while 0 indicates no overlap and 1 indicates complete overlap, which can be mathematically expressed as

$$Dice\;Similarity\;Score=\;\frac{2\times True\;Positive}{2\times True\;Positive+False\;Positive+False\;Negative\;}.$$

The average surface distance is calculated as the average of all the distances from points on the boundary from model prediction to the ground truth boundary. We also measure the success rate of the *virtual dissection* algorithm, where a heart model is successfully parsed if the Intersect over Union (IoU) between the algorithm prediction and expert manual annotation is larger than 0.5. This metric has been widely used for detection tasks (42).

We expressed continuous data by mean ± SD and categorical data by percentages. The distance and Dice scores were summarized as medians and interquartile range (IQR). Pearson correlation's test was used to assess the similarity of LA volumes and LA sphericity Index estimated from model prediction and ground truth. Student's *t*-test, Chi-square test, or McNemar's test was applied as appropriate. *p* < 0.05 was considered as significant.

## 3. Results

### 3.1. Study populations

The demographics of the development, internal test and prospective cohorts are shown in Table 1. There were no statistical differences in demographics between cohorts except for a higher incidence of diabetes mellitus in the *Development* vs. *Internal Test* cohorts.

**Table 1:** Clinical demographics of retrospective and prospective study.

	Retrospective cohort (*N* = 130)			Prospective cohort (*N* = 42)	*p*-value	
	Entire cohort (*N* = 130)	Development cohort (*N* = 30)	Internal test cohort (*N* = 100)		Development vs. internal test	Development vs. prospective
Age, year, mean ± SD	64.8 ± 9.8	65.6 ± 10.1	64.6 ± 9.7	65.2 ± 10.6	0.64	0.88
Male gender, *N* (%)	95 (73.1%)	21 (70.0%)	74 (74.0%)	24 (57.1%)	0.39	0.05
Non-paroxysmal AF, *N* (%)	69 (53.1%)	16 (53.3%)	53 (53.0%)	18 (42.9%)	0.97	0.38
BMI, kg/m^2^, mean ± SD	29.7 ± 5.7	30.5 ± 5.0	29.43 ± 5.9	29.6 ± 6.9	0.33	0.52
Diabetes, N (%)	16 (12.3%)	7 (23.3%)	9 (9.0%)	5 (11.9%)	0.04	0.20
CHA2DS2-VASc score, mean ± SD	2.2 ± 1.4	2.3 ± 1.2	2.2 ± 1.8	2.1 ± 1.3	0.38	0.37
Smokers, *N* (%)	51 (39.2%)	16 (53.3%)	35 (35.0%)	14 (33.3%)	0.08	0.07
Enlarged LA, *N* (%)	77 (59.2%)	19 (63.3%)	58 (58.0%)	19 (45.2%)	0.60	0.13

### 3.2. Virtual dissection can automatically parse cardiac geometry

In digital hearts, our developed virtual dissection approach separated the PVs and LAA from left atrial bodies (Figure 3A) with a mean difference for the centroid-boundary distances of −0.27 mm (95% CI: −3.87–3.33; *r* = 0.99; *p* < 0.0001; Figure 3B). We randomly selected 5 shells of seed data from the *Development* cohort for tuning, with LA sizes from 71 to 140 ml that cover a broad range of patients (43).

In our *Internal Test* cohort (*N* = 100), we compared the optimized-virtual dissection to expert annotation using commercially available software (EnSite Verismo Segmentation Tool; Abbott/St Jude Medical, Inc., St. Paul, Minnesota) refined using 3D Slicer (41). Figure 3E shows the success rate of dissection. Accuracy increased from 67% (no tuning) to 94% by tuning with *N* = 5 shells of seed data (*p = *0*.*034; McNemar's test), then showed only modest changes when tuning in 10–30 shells (92%–94%). Tuned with *N* = 5 seed data, virtual dissection produced mean difference and limits of agreement for the centroid-boundary distance of 1.46 mm (95% CI: −5.58–8.49; *r* = 0.99; *p* < 0.0001; Figure 3D). Figure 3C presents two virtually dissected (left) and manually annotated (right) atria.

### 3.3. ML trained by virtual dissection can accurately segment CT

**Figure 4:**
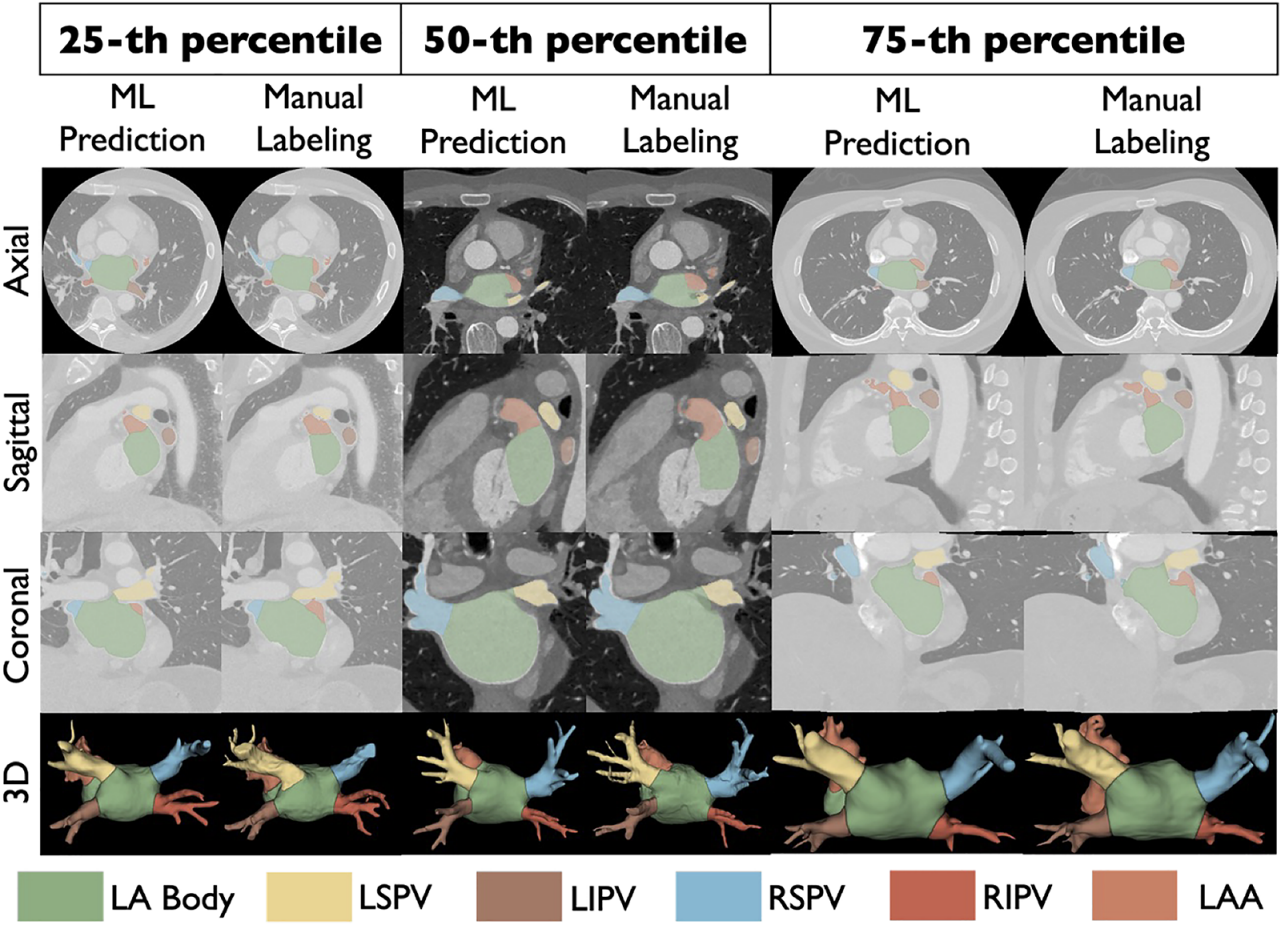
Comparison between the ML model predicted CT segmentation (left) and ground truth manual outlining (right) overlaid on the input CT scans in representative samples selected using 25th, 50th and 75th percentiles of segmentation accuracy in an independent test cohort (*N* = 100). Our ML model effectively captured the LA geometry, highlighting key features of PVs, LAA, and their ostia. The mitral valve plane represented in the 50th- and 25th- percentile samples showed slight variation between ML prediction and manual labeling, likely from limited image quality. Slight differences in PV measurements were found in the 25th-percentile sample, which may not be clinically relevant. Acronyms: LA, left atrium; LSPV, left superior pulmonary vein; LIPV, left inferior pulmonary vein; RSPV, right superior pulmonary vein; RIPV, right inferior pulmonary vein; LAA, left atrial appendage.

**Figure 5:**
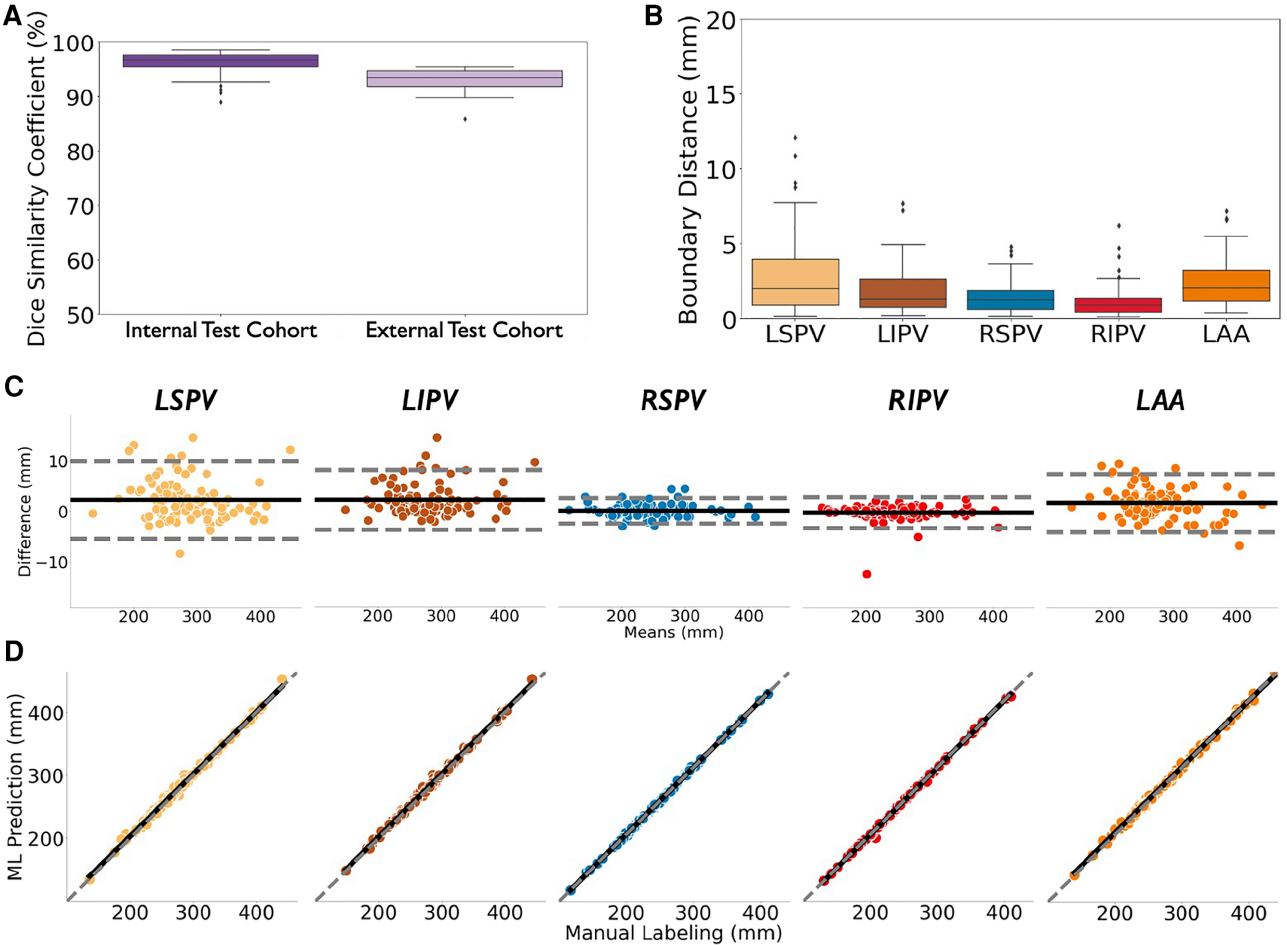
Accuracy CT segmentation using ML of optimized virtual dissection in two test cohorts (**A**) dice score of ML-based CT segmentation in the internal test cohort (*N* = 100; left) and an external test cohort from a different institution with different CT scanners (*N* = 60; right). (**B**) Boundary surface distances between ML-prediction and expert labelling in the Test Dataset (*N* = 100). (**C**) and (**D**) are Bland–Altman plots and linear regression plots of the centroid-to-boundary distance in the Test Dataset (*N* = 100). Acronyms: LSPV, left superior pulmonary vein; LIPV, left inferior pulmonary vein; RSPV, right superior pulmonary vein; RIPV, right inferior pulmonary vein; LAA, left atrial appendage.

Figure 4 shows comparisons between ML prediction (left) and manually labeled (right) atria from select samples, representing the 25th, 50th, and 75th percentile accuracy in the hold out *Internal Test* cohort (*N* = 100). Our ML model revealed LA structure, and successfully captured the shape and details of PVs, LAA, and their ostia. The mitral valve plane in the 50th- and 25th-percentile samples showed slight qualitative inconsistencies between ML prediction and ground truth, possibly due to variations in image quality such as density of contrast. Slight differences in LSPV and RSPV measurements were found in the 25th-percentile sample, but the ostia position differences between ML and expert annotations are limited, with LA-LSPV and LA-RSPV boundary errors in a range of 3.54 mm and 0.49 mm, respectively; these differences may not be clinically relevant. Overall, Dice scores were 96.7% (IQR: 95.3% – 97.7%) (Figure 5A, left), a median error in surface distance of boundaries of 1.51 mm (IQR: 0.72 – 3.12)) (Figure 5B) with a mean boundary distance of 1.16 mm (95% CI: −4.57–6.89) again similar to experts (r = 0.99; *p* < 0.001, Figure 5C-D).

In our External Test cohort (*N* = 60) of patients from another Institution with different scanners (10), the model segmented structures with a Dice score of 93.5% (IQR: 91.9% to 94.7%) (Figure 5A, right) again comparing favorably to experts (r = 0.99; *p* < 0.0001).

Thus, this approach enabled a > 10-fold reduction in the relative size of training to test cases for ML, inverting the ratio of training: test cases less than 1:3, from a typical ratio of >3:1.

### 3.4. Analysis of Anatomical Variants

Despite not pre-screening to eliminate anatomic variants, segmentation accuracy from our virtual dissection technique was maintained for variant anatomy. Overall, 100% cases with 4 PV ostia (the most common configuration, representing 66 cases) were parsed with mean difference and limits of agreement for the centroid-boundary distance of 1.26 mm (95% CI: −5.15–7.68; *r* = 0.99; *p* < 0.0001). We identified 29 cases with one of the 3 main variants: (1) common left PV ostia (*N* = 8; Supplementary Figure 2A); (2) LAA occlusion by a closure device (*N* = 1; Supplementary Figure 2B); and (3) supplemental PVs or ostial-branch PV (*N* = 20; Supplementary Figures 2C,D,G,H). The remaining 5 cases have a combination of these 3 main variants (Supplementary Figures 2E,F).

In summary, 28/34 of identified variants were successfully parsed with anatomic agreement within 1.95 mm (95% CI: −6.34–10.25) which again was in line with experts (*r *= 0.99; *p* < 0.0001), despite lack of specific training in variants. In the remaining 6 cases, errors arose mostly from missing PVs or branches relative to the 4 PV mathematical model (Figure 2A-1), which could be addressed by geometric models that adapt to a range of PVs.

### 3.5. Prospective validation: using virtual-dissection trained ML to segment left atria

**Figure 6:**
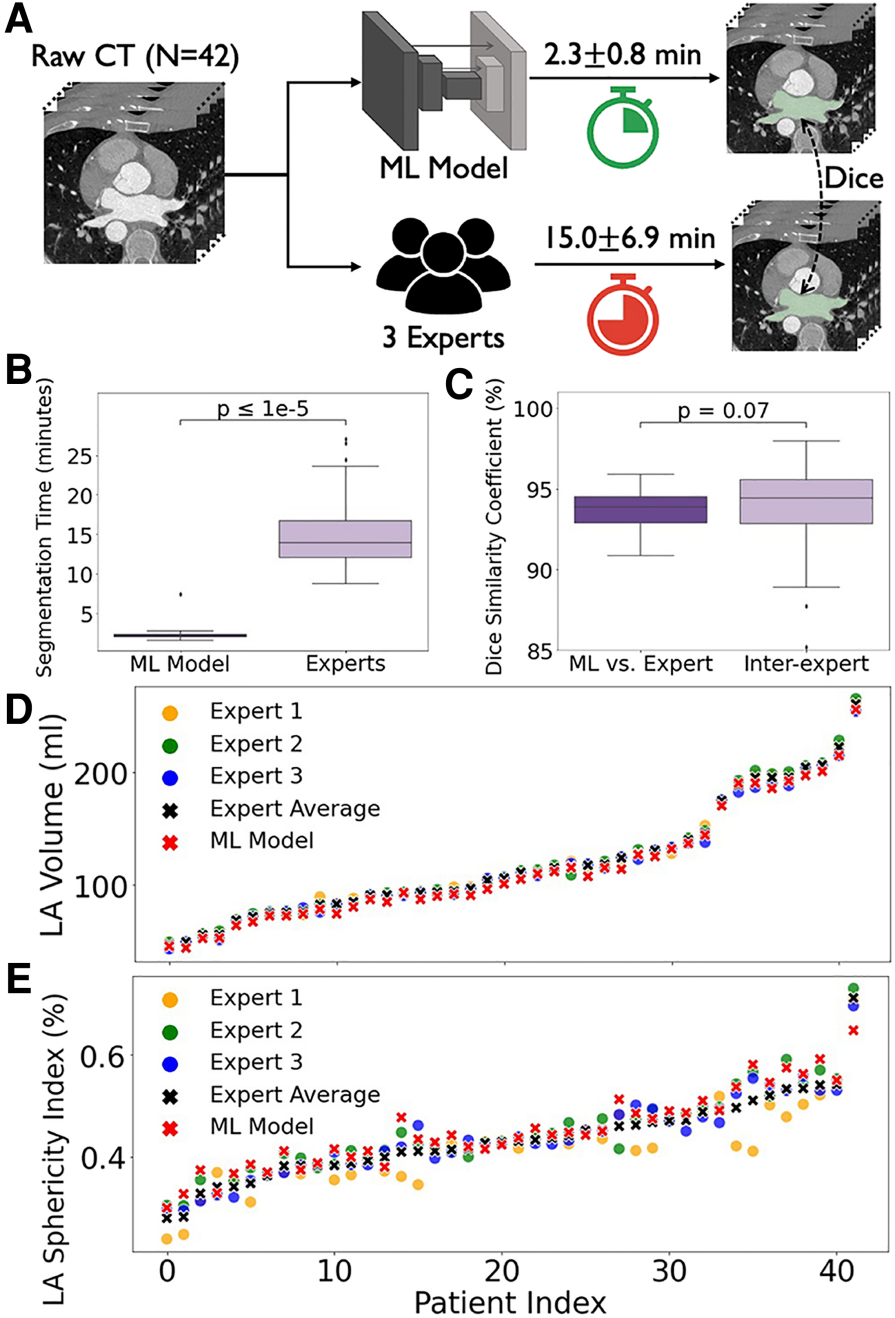
Prospective segmentation of cardiac CT scans in 42 consecutive patients undergoing AF ablation by virtual-dissection trained ML vs. experts. (**A,B**) Virtual dissection trained ML significantly shortens segmentation time compared to experts. (**C**) Box plots of Dice similarity coefficient between ML and experts were similar. (**D**) and (**E**) LA volume and LA sphericity index marked by Virtual Dissection (red cross) accurately tracks the mean between experts (black cross).

Prospectively, in patients prior to AF ablation, the ML model shortened mean left atrial/PV segmentation time by 85.0% compared to the expert panel (2.3 ± 0.8 vs. 15.0 ± 6.9 min, *p* < 0.0001; Figures 6A,B). Figure 6C shows that our model achieved a Dice score of 93.9% (IQR: 93.0%–94.6%) compared to a panel of 3 experts, statistically indistinguishable from the inter-observer agreement between experts of 94.4% (IQR: 92.8%–95.7%, *p* = 0.071).

To further analyze CT segmentation by our geometrically encoded ML, we compared the left atrial volume and sphericity index between ML and expert readings. These indices are well reported measures of abnormal left atrial size and shape that predict recurrence of AF after drug therapy or ablation (44, 45). Figures 6D,E shows that they were well correlated (*r* = 0.99 and 0.95, respectively; *p < *0*.*0001).

## 4. Discussion

Mathematical encoding of geometry was able to accelerate ML for segmentation of CT, and enable its training on very small datasets. In our study, the training: testing ratio was <1 training to 3 test, which indicates a far lower need for training than the conventional published ratios of >3:1 for ML (11–15). This “inversed training-test ratio” paradigm has recently been applied in domains outside medicine such as for Amazon co-purchasing product predictions (46). Our approach was then tested in two independent test datasets and in a prospective study prior to AF ablation, in which the model accelerated segmentation while maintaining similar accuracy to experts. This novel approach could broaden the ease of access and accuracy of AF ablation. More broadly, this approach has analogies to natural intelligence, which has the potential to reduce the need for large annotated datasets to train ML, and could be applied for diverse imaging applications.

### 4.1. Benefits for clinical applications

Cardiac CT is increasingly used (12, 14, 47) to guide ablation forAF, and to predict clinical endpoints such as the risk of AF recurrence (45, 48). However, segmentation of these large 70–200 MB datasets manually by experts may take up to tens of minutes (9–12) even with the latest commercial software (49, 50). Our prospective validation demonstrated ML models reduced segmentation time by 10–15 min, representing a reduction of 15%–20% from reported PVI case times of 60–100 min (51, 52), and a larger reduction compared to some recently reported segmentation times of 60–120 min (9–12).

Additionally, existing cardiac mapping systems like Carto® 3 (3) require manual input, and their segmentation varies based on the operator's skill and experience. In contrast, our approach offers a fast and fully automatic solution with ensured consistency. It also allows for manual review and editing if desired.

Moreover, our automated segmentation approach provides an efficient way to label and collect large databases—a feature not available in current cardiac mapping systems like Carto® 3 and Rhythmia, which store data in proprietary formats that are not readily accessible to researchers, and require manual input which hinders scalability.

### 4.2. Comparison to other studies for ML segmentation of cardiac anatomy

We compared our approach with 4 recent ML studies using traditional large training datasets to segment LA from CT scans (12–15). Baskaran et al. and others (12–15) trained in 73–230 cases using manual segmentations, and only tested in 17–37 cases with a maximum Dice of 95.6% (14). Our model used 3–10 times fewer training data yet outperformed on a test set 3–5 times larger. Our model also showed superior generalizability in external and prospective test cohorts, not included in (12–15).

Our approach circumvents the limitation that most CT studies that segmented the LA often did not specifically segment the PVs and LAA (12, 14). Our approach can accurately reveal other anatomical landmarks, evidenced by our ML model's high Dice score (96.7%) compared to experts. Supplementary Figure 3 illustrates that our ML model successfully identifies the roof and septal walls, which play a significant role in cardiac mapping and AF ablation procedures (53, 54). Our approach can also be applied to other cardiac imaging applications including segmentation of Magnetic Resonance Imaging (MRI) to boost ML by reducing the need for large training data sets.

### 4.3. Limitations

This study has several limitations. We used only CT and, although this is by far the most widely applied cardiac imaging modality, future studies could extend our approach to MRI through transfer learning. While we tested our approach in cohorts from different institutions, additional studies are needed to define its sensitivity to data from a wide variety of scanners and spatial resolutions. We focused on improving left atrial segmentation, because it is an important and common clinical task, but future studies should extend to other features such as segmenting CT scans to study the aorta for aneurysmal dilation (55), which has a high mortality rate (56), or to plan aortic valve replacement (57), which is commonly performed (58). One limitation and future direction for this work is to adapt our domain knowledge encoding algorithm for different variants, including but not limited to a range of PVs, or congenital variants in the ventricles or aorta (57).

## 5. Conclusion

Domain knowledge encoding of cardiac geometry was able to train Machine Learning to segment cardiac CT while greatly reducing the need for large training data sets. Our approach (virtual dissection) derived in very small datasets was able to accurately segment cardiac CT in 2 independent datasets of hundreds of patients and in a prospective study prior to AF ablation. In general, this combined domain knowledge encoding and machine learning approach reduce the dependence of ML on large training datasets and could be applied broadly in imaging and benefit personalized cardiovascular medicine.

## Data Availability

The 3D digital heart models can be found in online repository: https://zenodo.org/record/4309958#%23.YdlOJRPMJqs. The external dataset MM-WHS can be publicly accessed at http://www.sdspeople.fudan.edu.cn/zhuangxiahai/0/mmwhs/. The remaining datasets presented in this article are not readily available because the internal datasets for retrospective and prospective studies are not currently permitted for public release due to the sensitive nature of patient data. Requests to access the datasets should be directed to RF, ruibin@stanford.edu.
