# Supplementary material for: Segmenting computed tomograms for cardiac ablation using machine learning leveraged by domain knowledge encoding
Source: Front Cardiovasc Med. 2023 Oct 2;10:1189293. doi: 10.3389/fcvm.2023.1189293 (PMC10577270; doi:10.3389/fcvm.2023.1189293)
Supplement: Supplementary file 1 [file Datasheet1.pdf]

Supplementary Material

Segmenting Computed Tomograms for Cardiac Ablation Using Machine Learning Leveraged by Domain Knowledge Encoding

Ruibin Feng, PhD, Brototo Deb, MD, Prasanth Ganesan, PhD, Fleur V.Y. Tjong, MD, PhD, Albert J. Rogers, MD, Samuel Ruipérez-Campillo, MS, MEng, Sulaiman Somani, MD, Paul Clopton, MS, Tina Baykaner, MD, Miguel Rodrigo, PhD, James Zou, PhD, Francois Haddad, MD, Matei Zahari, PhD, Sanjiv M. Narayan*, MD, PhD

*** Correspondence:** Sanjiv M. Narayan, MD, PhD: sanjiv1@stanford.edu

1 Supplementary Methods

1.1 Machine Learning Model Training Protocol

To train our ML model **(**Supplemental Figure 1**)**, we first z-score normalized each input CT scan by subtracting its mean, followed by division with its standard deviation. Then the images are re-sampled using third-order spline interpolation. The target voxel spacing is set as the median spacing of the training samples. To improve the generalizability, a set of data augmentation techniques were randomly applied on the fly during training, including rotations, flipping, scaling, Gaussian noise and blur, and random changes of brightness, contrast, and gamma. During the training process, we set the batch size to 2 due to the GPU memory limitation and trained the DL model for 1000 epochs. Stochastic gradient descent (6) was used to optimize the model. The initial learning rate and Nesterov momentum were set to 0.01 and 0.99, respectively. We use the sum of cross-entropy and Dice loss as training loss. Supplemental Figure 4 shows the convergence of training loss, validation loss, and validation accuracy (measured by Dice) during training.

2 Supplementary Figures

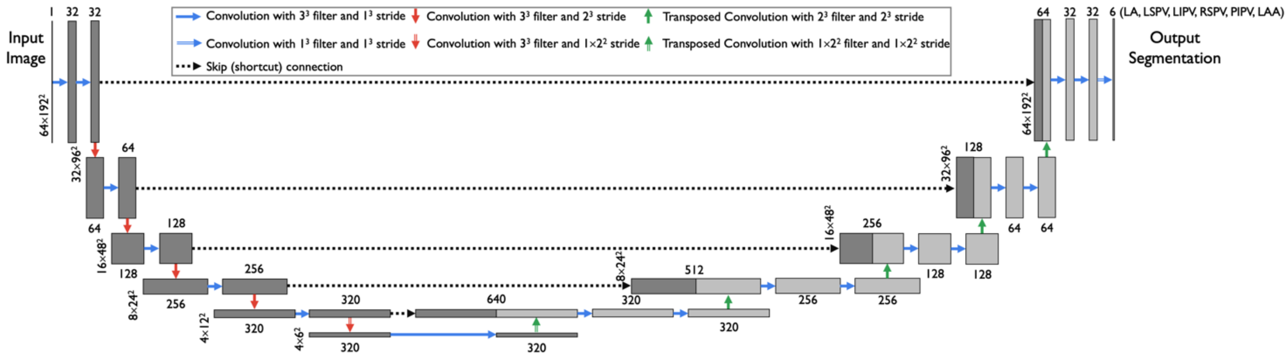

Supplemental Figure 1. Neural network architecture used for segmentation, based on nnU-Net (10). The vertical and horizontal numbers indicate the pixel and feature dimensions at each layer, respectively. All convolution and transposed convolution layers (except the last one) are followed by one instance normalization layer and one LeakyRelu layer, which are not illustrated in the figure.

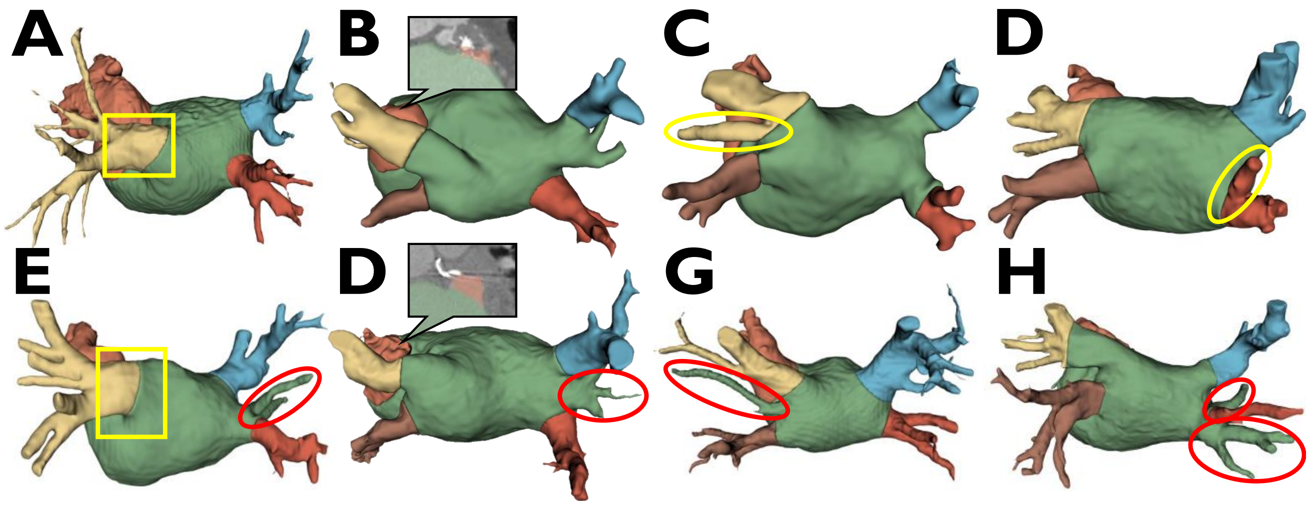

Supplemental Figure 2. Robust segmentation of anatomical variants by our virtual dissection algorithm, despite the fact that the algorithm was not designed or tuned specifically to handle them. The primary analysis identified 29 cases with one of the 3 main variants: (A) common left PV ostia (N = 8; yellow-boxed); (B) LAA occlusion by a closure device (N=1; the closure devices were identified on the CT images); (C-D) and (G-H) supplemental PVs or ostial-branch PV (N=20; the parsed and missing PVs or branches are marked with yellow and red circle, respectively). There are 5 cases that have a combination of these 3 main variants. For example, (E) showed both common left PV ostia and ostial-branch PV, while (F) had LAA occlusion and a supplemental PV. Our algorithm was able to segment 28/34 of identified variants with these identified variants, while the errors arose mostly from missing PVs or branches (E-H; red circled).

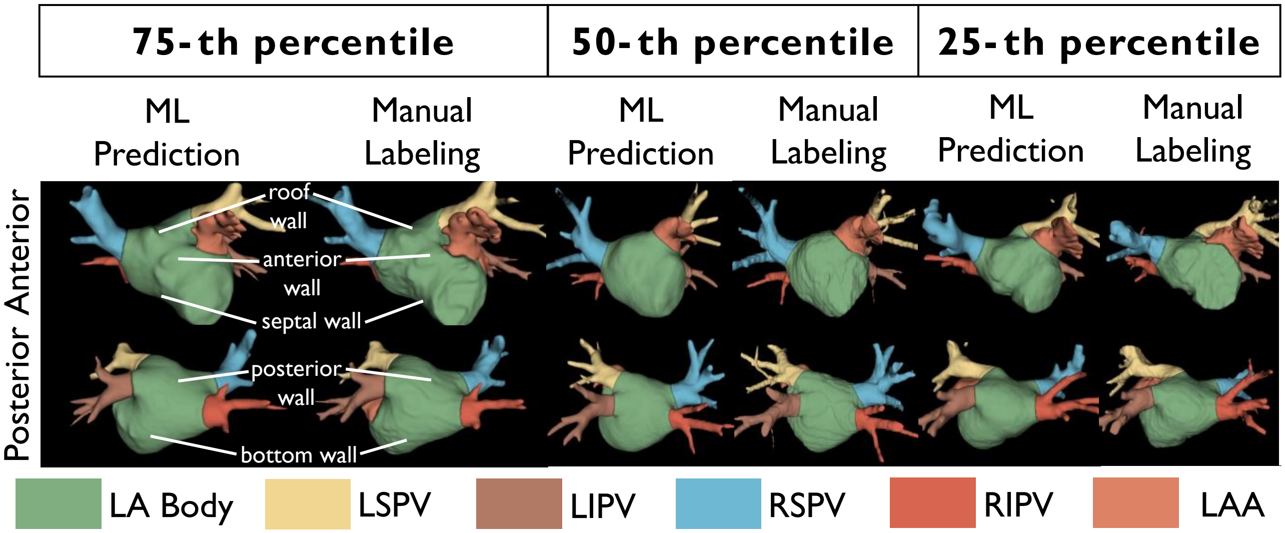

Supplemental Figure 3. Comparison between the ML model predicted CT segmentation (left) and ground truth manual outlining (right) overlaid on the input CT scans in representative samples selected using 75-th, 50-th, and 25-th percentile of segmentation accuracy in an independent Test cohort (N=100). Both anterior (top) and posterior (bottom) views are provided. Our model can accurately reveal anatomical landmarks, including roof, anterior, septal, posterior, and bottom walls. Acronyms: LA: Left Atrium, LSPV: Left Superior Pulmonary Vein, LIPV: Left Inferior Pulmonary Vein, RSPV: Right Superior Pulmonary Vein, RIPV: Right Inferior Pulmonary Vein, LAA: Left Atrial Appendage.

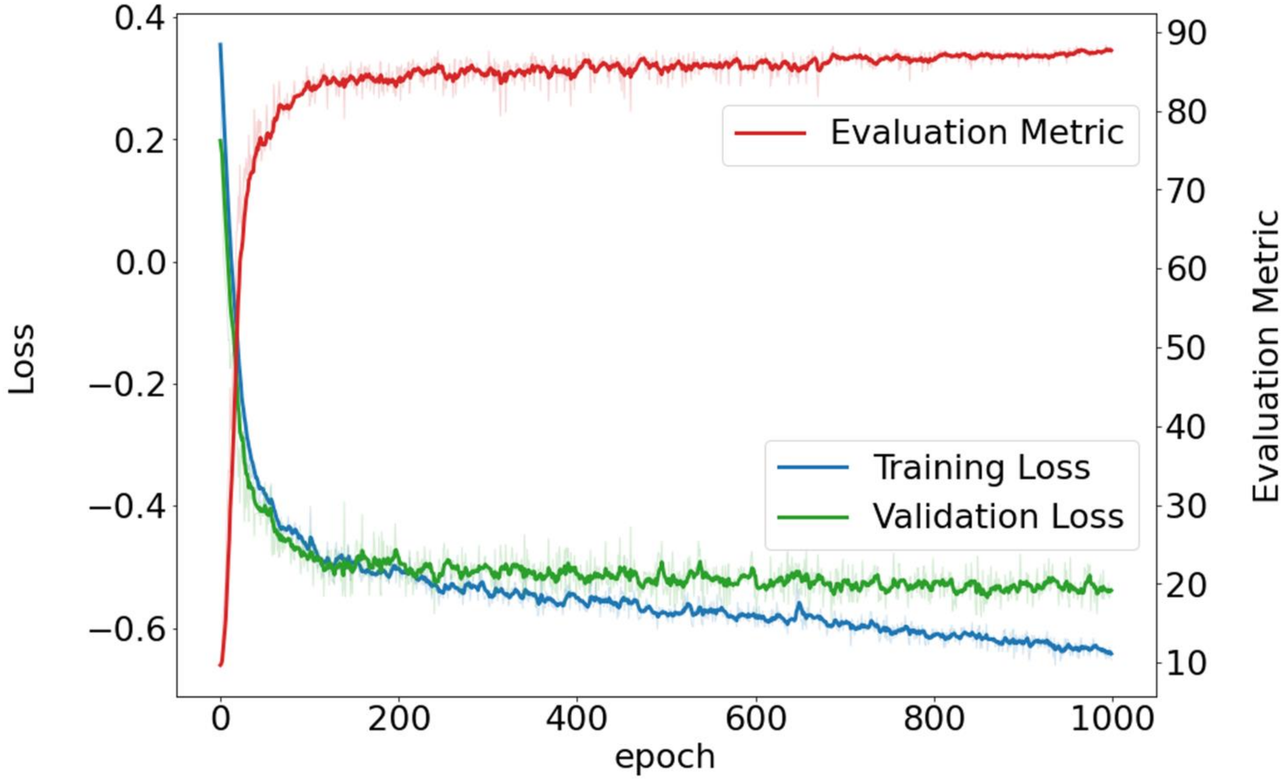

Supplemental Figure 4. The convergence of training loss, validation loss, the validation accuracy (measured by Dice) during training.

Reference

1. Fedorov A, Beichel R, Kalpathy-Cramer J, Finet J, Fillion-Robin J-C, Pujol S, et al. 3d Slicer as an Image Computing Platform for the Quantitative Imaging Network. *Magnetic resonance imaging* (2012) 30(9):1323-41.

2. Tobon-Gomez C, Geers AJ, Peters J, Weese J, Pinto K, Karim R, et al. Benchmark for Algorithms Segmenting the Left Atrium from 3d Ct and Mri Datasets. *IEEE transactions on medical imaging* (2015) 34(7):1460-73.

3. Krissian K, Carreira JM, Esclarin J, Maynar M. Semi-Automatic Segmentation and Detection of Aorta Dissection Wall in Mdct Angiography. *Medical image analysis* (2014) 18(1):83-102.

4. Razeghi O, Sim I, Roney CH, Karim R, Chubb H, Whitaker J, et al. Fully Automatic Atrial Fibrosis Assessment Using a Multilabel Convolutional Neural Network. *Circulation: Cardiovascular Imaging* (2020) 13(12):e011512.

5. Piccinelli M, Veneziani A, Steinman DA, Remuzzi A, Antiga L. A Framework for Geometric Analysis of Vascular Structures: Application to Cerebral Aneurysms. *IEEE transactions on medical imaging* (2009) 28(8):1141-55.

6. Ketkar N. Stochastic Gradient Descent. *Deep Learning with Python*. Springer (2017). p. 113-32.

7. Ho SY, Cabrera JA, Sanchez-Quintana D. Left Atrial Anatomy Revisited. *Circulation: Arrhythmia and Electrophysiology* (2012) 5(1):220-8.

8. Scharf C, Sneider M, Case I, Chugh A, Lai SW, Pelosi Jr F, et al. Anatomy of the Pulmonary Veins in Patients with Atrial Fibrillation and Effects of Segmental Ostial Ablation Analyzed by Computed Tomography. *Journal of cardiovascular electrophysiology* (2003) 14(2):150-5.

9. Chen X, Fang P, Liu Z, He J, Tang M, Liu J, et al. Pulmonary Vein Anatomy Is Associated with Cryo Kinetics During Cryoballoon Ablation for Atrial Fibrillation. *Arquivos Brasileiros de Cardiologia* (2018) 110:440-8.

10. Isensee F, Jaeger PF, Kohl SA, Petersen J, Maier-Hein KH. Nnu-Net: A Self-Configuring Method for Deep Learning-Based Biomedical Image Segmentation. *Nature methods* (2021) 18(2):203-11.
